# Emission of direct-gap band in germanium with Ge-GeSn layers on one-dimensional structure

**DOI:** 10.1038/srep24802

**Published:** 2016-04-21

**Authors:** Zhong-Mei Huang, Wei-Qi Huang, Shi-Rong Liu, Tai-Ge Dong, Gang Wang, Xue-Ke Wu, Cao-Jian Qin

**Affiliations:** 1 State Key Laboratory of Surface Physics, Key Laboratory of Micro and Nano Photonic Structures (Ministry of Education) and Department of Physics, Fudan University, Shanghai 200433 (China); 2 Institute of Nanophotonic Physics, Guizhou University, Guiyang 550025 (China); 3 State Key Laboratory of Environmental Geochemistry Institute of Geochemistry, Chinese Academy of Science Institute of Geochemistry, Guiyang 550003 (China)

## Abstract

In our experiment, it was observed that the emission of direct-gap band in germanium with Ge-GeSn layers on one-dimensional (1D) structure. The results of experiment and calculation demonstrate that the uniaxial tensile strain in the (111) and (110) direction can efficiently transform Ge to a direct bandgap material with the bandgap energy useful for technological application. It is interested that under the tensile strain from Ge-GeSn layers on 1D structure in which the uniaxial strain could be obtained by curved layer (CL) effect, the two bandgaps E_Γg_ and E_Lg_ in the (111) direction become nearly equal at 0.83 eV related to the emission of direct-gap band near 1500 nm in the experiments. It is discovered that the red-shift of the peaks from 1500 nm to 1600 nm occurs with change of the uniaxial tensile strain, which proves that the peaks come from the emission of direct-gap band.

Germanium is a material with special properties such as high mobility of electrons and holes. But Ge is an indirect bandgap material, so an electron transits from the conduction band to the valence band mainly by phonon-assisted recombination with non-radiation process, which results in slow and inefficient direct optical recombination. It is desirable to obtain a direct bandgap for Ge, in which some results have been achieved in two main ingredients at the origin of the enhancement of the optical recombination properties of germanium: *n*-type doping and tensile strain^1^,^2^,^3^. Recent, germanium is attracting a considerable interest due to the possibility to achieve direct-bandgap emission with this material on a silicon wafer^4^,^5^. Germanium has been the subject of considerable attention as a candidate in the integrated laser on silicon platforms^6^.

Although germanium is an indirect-bandgap semiconductor with the conduction band minimum located at the L point, a local minimum is present at Γ point in the conduction, so manipulating the band structure through strain and doping could enable efficient radiation recombination. If a higher tensile strain is applied to germanium crystal, the induced deformation can introduce a crossover of the Γ minima and L minima in conduction to convert germanium into a direct-bandgap semiconductor^7^. H Tahini *et al*.^8^ investigated using DFT uniaxial strain-induced changes from the indirect to direct bandgap transition as a function of strain in Ge. In the letter, our experiments demonstrate that the uniaxial tensile strain in the (111) and (110) direction originated from Ge-GeSn layers on one-dimensional (1D) structure, in which the uniaxial strain could be obtained by curved layer (CL) effect, can efficiently transform Ge to a direct bandgap material with the bandgap energy useful for technological application.

The silicon wafers of P-type substrate with 1–10 Ωcm were taken on the sample stage in the combination fabrication system with nanosecond pulsed laser etching (PLE) and pulsed laser depositing (PLD) devices as shown in Fig. 1^9^,^10^. A nanosecond pulsed Nd:YAG laser (wavelength: 1064 nm, pulse length: 60 ns FWHM, repetition rate: 1000) was used to etch the one-dimensional (1D) structures (such as pillar and cavity with linear shape) on Si chip in PLE process. On the Si 1D structure (pillar) as shown in the inset of Fig. 1, a third harmonic of pulsed Nd:YAG laser at 355 nm was used to deposit the Ge-GeSn layers in PLD process to obtain the uniaxial tensile strain (see Methods). The crystal structures of the Ge-GeSn layers on Si pillar prepared by PLD process after annealing can be obtained in the (110) and (111) direction^2^.

Figure 2 shows the forming mechanism of the uniaxial tensile strain in Ge-GeSn layers covering on the 1D structure, in which the uniaxial strain is kept along Z direction while the strain along S direction is relaxed (see Methods). It is called curved layer (CL) effect in the rolled-down layers (Fig. 2(a)) and the rolled-up layers (Fig. 2(b)) of GeSn-Ge structures covering on the 1D structures (linear pillar or linear cavity), respectively^11^, in which the difference ΔS of the arc length between GeSn layer center and Ge layer center should be equal to the mismatch of crystal lattice constant between GeSn and Ge (see Methods).

The photoluminescence (PL) spectra on the Ge-GeSn crystal structures in (111) and (110) direction with the uniaxial strain are measured under the 488 nm excitation by using PL measurement system at 17K (see Methods), in which the peak near 1500 nm related to the energy of 0.83 eV has been observed, as shown in Fig. 3(a). They have the emission characteristics of direct-gap band in germanium (such as the thresholds effect, the supper-linear or linear increase intensity effect with increasing pumping and the quenching effect at high temperature (>150K)). The experimental result demonstrates that the uniaxial tensile strain in the (111) and (110) direction can efficiently transform Ge to a direct bandgap material, which agrees with the results of simulation calculation as shown in Fig. 3(b)^8^. Here it is very interesting that the evolution of E_L_ valley point obviously rises with the uniaxial tensile strain in the (111) to keep the direct-gap valley point near 0.8 eV. Figure 3(c) shows the PL emission peak of Ge direct bandgap in the (111) direction, in which the inset shows the SEM image of 1D structure (linear cavity).

In Fig. 4, it is very interesting that the peak of direct bandgap emission near 1600 nm related to the uniaxial tensile strain in the (111) direction is obviously observed in the Ge-GeSn layers on the 1D structure as shown in the inset of Fig. 4, in which the diameter of the 1D structure (micro-nano-pillar) is about 100 nm and the thickness of the Ge-GeSn layers is about 10 nm, where the strain along S direction is almost relaxed according to ruler in CL effect for GeSn-Ge (see Methods).

It is very interesting that the peaks in the PL spectra have obviously red-shifting with larger strain measured in the Ge-GeSn layers on the 1D structure as shown in the SEM image of Fig. 5(a), in which the peak near 1500 nm shifts to 1663 nm, where the peak disappears in the Ge-Si layers on the 1D structure in (111) direction which indicates that GeSn plays a important role, as shown in Fig. 5(b). It is proved that the peak shift of direct bandgap emission is originated from the change of uniaxial strain on the 1D structure^12^,^13^, in which the diameter of the 1D structure (pillar) is about 500 nm and the thickness of the Ge-GeSn layers is about 50 nm where the strain along S direction is relaxed according to ruler in CL effect for GeSn-Ge: the mismatch is equal to △S when the thickness of the Ge-GeSn layers is about R/10 (R: Curvature radius of the 1D structure) as shown in Fig. 2 (see Methods). Figure 5(c) shows the change curves of PL intensity with rising temperature from 17K to 200K, in which it is noted that the peak at 1663 nm disappears (quench effect) at 150K obviously.

Figure 6(a) shows the Ge-GeSn crystal structure in (110) direction growing on the 1D structure prepared by PLD process and the linear cavity (the inset SEM image) prepared by PLE process. The PL spectrum on the Ge-GeSn crystal structure in (110) direction is measured under the 488 nm excitation by using PL measurement systems at 17K (see Methods), as shown in Fig. 6(c), in which the sharper peak near 1700 nm related to the energy of 0.73 eV has been observed. It has the emission characteristics of direct-gap band in germanium. The experimental result demonstrates that the uniaxial tensile strain in the (110) direction can efficiently transform Ge to a direct bandgap material, which agrees with the results of simulation calculation as shown in Fig. 6(b)^8^. Figure 6(c) shows the intensity evolution of PL peak near 1700 nm with increase of pumping power, which is originated from the direct-bandgap.

In Fig. 7, it is interesting that the PL peak has obviously red-shifting with larger strain measured in the Ge-GeSn layers on the 1D structure (pillar) as shown in the inset of SEM image, in which the peak near 1700 nm shifts to 1814 nm, where Sn plays a important role due to the peak disappearing (red curve) in the Ge-Si layers on the 1D structure.

We have chosen some model in order to simulate the experimental process. The electronic behavior is investigated in the work by an abinitio non-relativistic quantum mechanical analysis. The DFT calculation were carried out by using the local density approximation (LDA) and non-local gradient-corrected exchange-correlation functional (GGA) for the self-consistent total energy calculation. In the simulating calculation, Fig. 8 shows the Ge-Sn layers model (a and b) and its band structure (c), in which the valley of conduction band of germanium at L point rises to be higher than the valley of conduction band atΓpoint originated from the tensile strain of Ge-Sn layers on the 1D structures. The deformation of the Ge-Sn layers under the tensile strain is shown in Fig. 8(b) (after the optimum of structure) related to Fig. 8(a) (before the optimum of structure). Figure 8(b) shows that the deformation of Ge atom lattice is originated from the tensile strain in the germanium on the Ge-Sn layers, which results in valley rising of conduction band at L point as shown in Fig. 8(c).

Besides the strain induced from the mismatch of GeSn-Ge layers structure, the SnGe alloy with partially relaxing strain could also build a direct bandgap material with the bandgap energy useful for optical communication. Figure 9 shows the physics process of simulation, in which Fig. 9(a) describes the structure of SnGe alloy which makes the rising of theΓvalley to form the direct bandgap structure as shown in Fig. 9(b), and its density of states is shown in Fig. 9(c).

In the conclusion, we have fabricated the Ge-GeSn layers in the (111) and (110) direction on the 1D structure of silicon for getting uniaxial tensile strain, and measured their PL peaks near 1500 nm and 1700 nm at 17K which have the characteristics of the direct-gap band material with the band gap energy useful for technological application. In the experiments, it is interesting that the PL peaks have obviously red-shifting with change of uniaxial strain measured in the Ge-GeSn layers on the one-dimensional structure. The intensive emission near 1500 nm is observed in the Ge-SnGe layers on the 1D structure with SnGe alloy. It is a new way to obtain the emission of direct-gap band in four-group materials.

## Methods

### Preparation of Ge-GeSn layers

Some silicon wafers of P-type (100) oriented substrate with 1–10 Ωcm are taken on the sample stage in the combination fabrication system with pulsed laser etching (PLE) and pulsed laser deposition (PLD) devices. A pulsed Nd:YAG laser (wavelength: 1064 nm, pulse length: 60 ns FWHM, repetition rate: 1000) is used to etch 1D structures (pillar and linear cavity) on Si sample in PLE process. On the pillar or linear cavity, a third harmonic of pulsed Nd:YAG laser at 355 nm is used to deposit the Ge-GeSn (Alloy:5Ge/1Sn) layers on the 1D structures (roll-down layers on pillar or roll-up layers on linear cavity of silicon) in PLD process at 500 °C. After annealing and quenching or irradiation of electron beam, the biaxial tensile strain in germanium can be obtained on Ge-GeSn layers, and the uniaxial tensile strain in germanium can be obtained on curved layers of Ge-GeSn covering the 1D structures.

### Transmission electron microscope (TEM) analysis

The crystals in (111) and (110) direction on Ge-GeSn layers for TEM are made by PLD process after annealing and quenching. In the process there are several steps, the crystal structures of Ge-GeSn layers are prepared by PLD method at 500 °C, and then the crystal of Ge-GeSn layers in (111) or (110) direction is gradually formed after annealing and quenching. It is interesting that the crystal structures of Ge-GeSn layers can be built by using irradiation of electron beam^14^. In the TEM (JEM-2000FX) image, the crystal structures in (111) and (110) direction on Ge-GeSn layers were observed on the samples.

### Getting uniaxial tensile strain in germanium by CL effect

**Figure 2:**
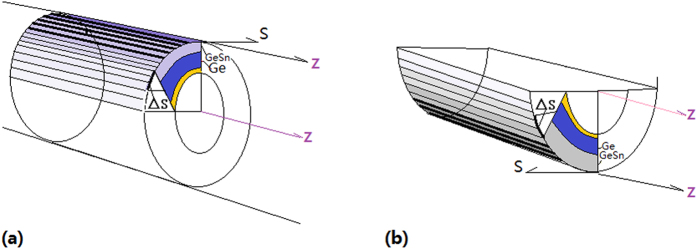
Forming mechanism of the uniaxial tensile strain in Ge-GeSn layers covering on the 1D structure (roll-down layers on pillar or roll-up layers on linear cavity).

At first, the one-dimensional (1D) structure of silicon is built on the silicon on insulator (SOI) substrate by using pulsed laser etching (PLE) and electron beam lithography (EBL) methods, then the Ge-GeSn layers are covered on the 1D structure (roll-down layers on pillar or roll-up layers on linear cavity of silicon) by using pulsed laser deposition (PLD) process, such as micro-nano-pillar of diameter 100 nm and Ge-GeSn layers of thickness 10 nm are fabricated, where the tensile strain along S direction is almost relaxed according to the ruler for GeSn-Ge: the mismatch of crystal constants between GeSn and Ge is equal to △S the difference of the arc lengths between layer centers of GeSn and Ge while the thickness of the Ge-GeSn layers is about R/10 (R: Curvature radius of the 1D structure), which is called curvature layer (CL) effect, as shown in Fig. 2.

### Photoluminescence (PL) measurement

PL spectra of the samples are measured under the 488 nm excitation at room temperature (300 K) and lower temperature (17~200 K) in sample chamber of 1Pa. In the PL measurement system, the light beam at 488 nm in Ar ion laser is focused on the samples for excitation.

### Annealing and quenching process

The samples are sent into the annealing furnace filled with Ar atmosphere to make annealing at 550 °C for 10 min or 15 min and quenching. The PL spectra show that annealing time of 15 min is suitable for crystallization and emission. In quenching process, the sample at 550 °C gotten out the annealing furnace is rapidly taken into water at room temperature for the sample to be rapidly cooled so that the sample structure at high temperature could be kept for solidification.

### Crystallizing under irradiation of electron beam

The amorphous silicon film was exposed under electron beam with 0.5 nA/ nm^2^ for 20~30 min in Tecnai G2 F20 system, in which electron beam from field‐emission electron gun, accelerated by 200 KV, has higher energy and better coherence. After irradiation under electron beam over 20 min, crystals of Ge-GeSn layers in (111) or (110) direction are gradually built.

## Additional Information

**How to cite this article**: Huang, Z.-M. *et al*. Emission of direct-gap band in germanium with Ge-GeSn layers on one-dimensional structure. *Sci. Rep.* **6**, 24802; doi: 10.1038/srep24802 (2016).

## Figures and Tables

**Figure 1:**
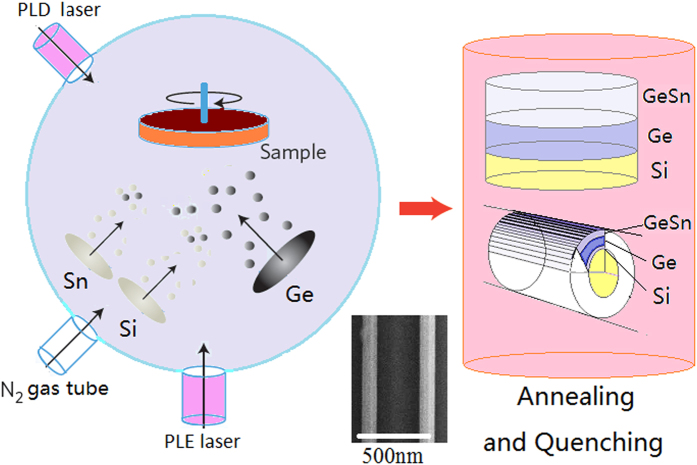
Fabrication system with PLE and PLD device, in which the inset shows the SEM image of the Ge-GeSn layers on 1D structure (pillar).

**Figure 3:**
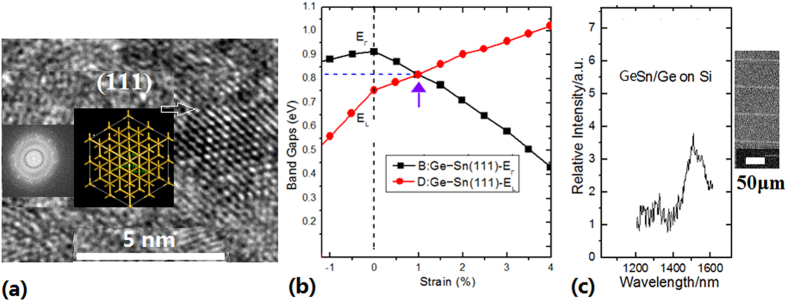
(**a**) TEM image of Ge-GeSn layers on silicon linear cavity prepared by PLD process, which shows crystal structure in (111) direction after annealing; (**b**) Evolution curves of energy bandgap with change of uniaxial tensile strain in the (111) direction in the calculation results^6^; (**c**) PL spectrum at 17 K in the Ge-GeSn layers on silicon linear cavity (as shown in the inset) prepared by PLD process after annealing, in which the peak occurs near 1500 nm.

**Figure 4:**
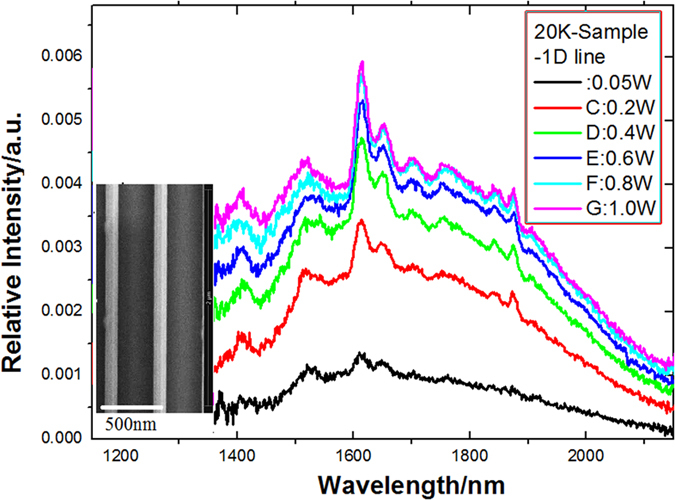
Intensity evolution with pumping change of power in the PL spectra at 20 K, in which the peaks of direct bandgap emission near 1600 nm related to the uniaxial tensile strain in the (111) direction are obviously observed in the Ge-GeSn layers on the 1D structure (linear pillar as shown in the inset).

**Figure 5:**
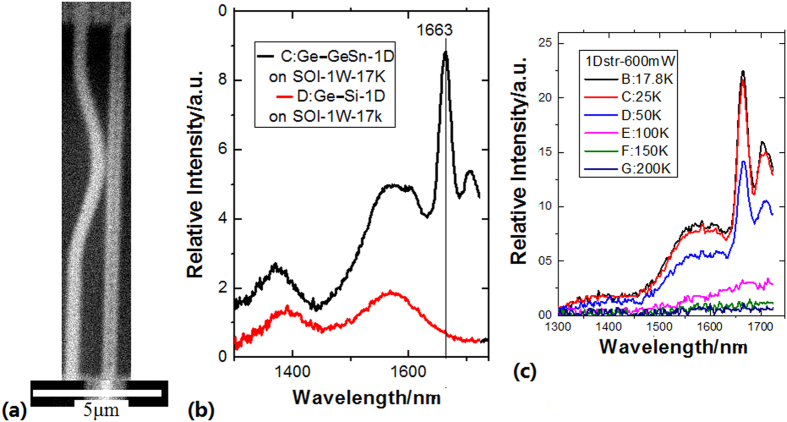
(**a**) SEM image of silicon linear pillar on which Ge-Si layers are deposited roll-down; (**b**) The peak of direct bandgap emission near 1663 nm related to the uniaxial tensile strain in the (111) direction in the PL spectra measured in the Ge-GeSn layers on the 1D structure (pillar), in which emission near 1663 nm is not observed in the Ge-Si layers on the 1D structure; (**c**) Change curves of PL intensity with rising temperature from 17 K to 200 K, in which it is noted that the peak at 1663 nm disappears at 150 K obviously.

**Figure 6:**
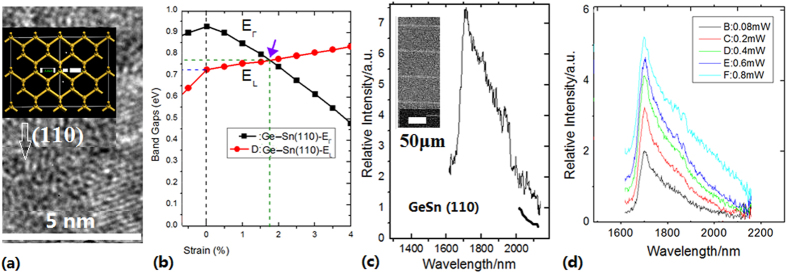
(**a**) TEM image of Ge-GeSn crystal in (110) direction after annealing on silicon pillars prepared by PLD process; (**b**) Evolution curves of energy bandgap with change of uniaxial tensile strain in the (110) direction in the calculation results^6^; (**c**) PL peak near 1700 nm measured at 17 K in the Ge-GeSn layers on silicon pillar (as shown in the inset) prepared by PLD process after annealing; (**d**) Intensity evolution with pumping change of power, in which the peaks occur near 1700 nm.

**Figure 7:**
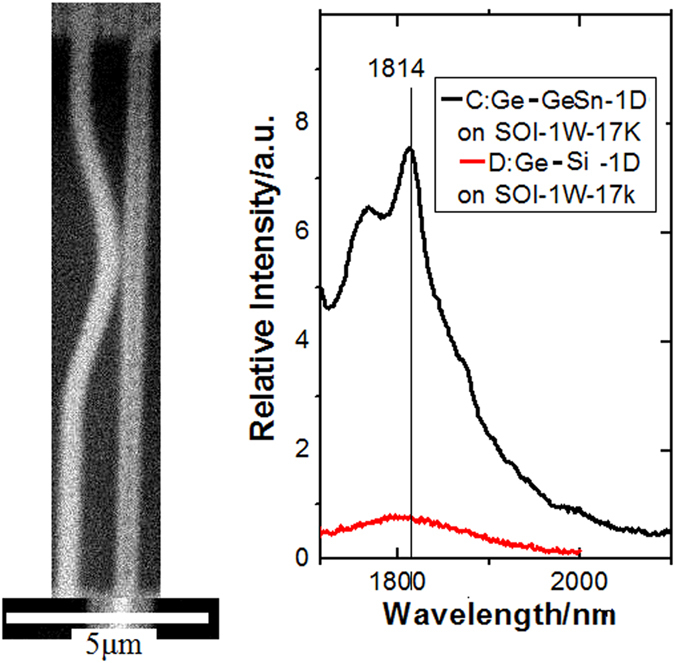
The peak of direct bandgap emission near 1800 nm related to the uniaxial tensile strain in the (110) direction obviously observed in the PL spectra measured in the Ge-GeSn layers on the 1D structure, in which no emission near 1800 nm is obviously observed in the Ge-Si layers on the 1D structure.

**Figure 8:**
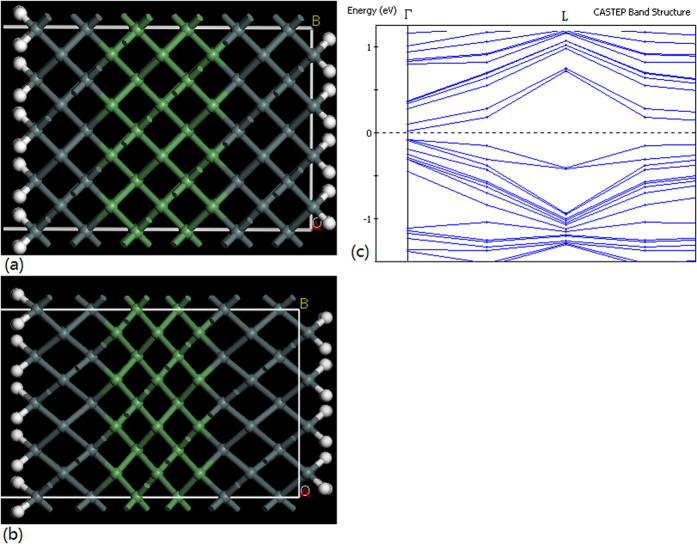
Simulating calculation and experimental measurement of the tensile strain on Ge-Sn layers. (**a**) Ge-Sn layers under no-strain condition (**b**) Ge-Sn layers under the tensile strain condition (**c**) Energy band structure in Ge-Sn layers under the tensile strain which makes a direct-gap band structure.

**Figure 9:**
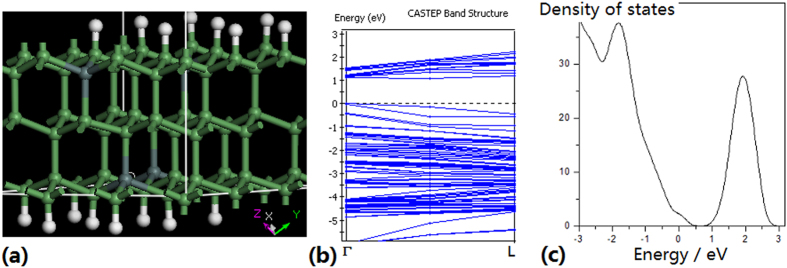
Physics process of simulation. (**a**) Structure of SnGe alloy (**b**) Band structure in the structure of SnGe alloy which makes the rising of the Γ valley to form the direct bandgap structure (**c**) Density of states in the structure of SnGe alloy.
